# Pet owners’ perspectives on veterinary biobanking in Latvia: Awareness, motivations, ethical concerns, and willingness to participate

**DOI:** 10.14202/vetworld.2025.3162-3173

**Published:** 2025-10-26

**Authors:** Gundega Stelfa, Kaspars Kovalenko, Liga Kovalcuka

**Affiliations:** 1 Clinical Institute, Faculty of Veterinary Medicine, Latvia University of Life Sciences and Technologies, K. Helmana 8, Jelgava, LV-3004, Latvia; 2 Institute of Food and Environmental Hygiene, Faculty of Veterinary Medicine, Latvia University of Life Sciences and Technologies, K. Helmana 8, Jelgava, LV-3004, Latvia

**Keywords:** comparative medicine, One Health, pet owners, public attitudes, translational research, veterinary biobank

## Abstract

**Background and Aim:**

Veterinary biobanking advances translational research, companion animal health, and the ethical reuse of samples. Its success depends on public engagement and the pet owners’ willingness to contribute biological samples. However, awareness and attitudes toward veterinary biobanking remain largely unexplored in the Baltic region. This study aimed to assess Latvian pet owners’ awareness, willingness to donate, motivations, and concerns regarding veterinary biobanking, and to identify demographic and professional factors influencing participation.

**Materials and Methods:**

A cross-sectional online survey was distributed through social media and veterinary clinics across Latvia between April and May 2025. The questionnaire included 49 items covering awareness, willingness to donate, motivations, concerns, and demographics. Data from 164 pet owners were analyzed using descriptive statistics, the Chi-square tests with Bonferroni correction, and logistic regression.

**Results:**

Only 22% of respondents had prior awareness of veterinary biobanks. Despite this, 76% were willing to donate samples if their pet was seriously ill, and 67% even if their pet was healthy. Motivations included altruism (helping other animals), supporting veterinary research, and potential treatment benefits for their own pets. Key concerns centered on confidentiality (91%), the right to withdraw samples (60%), and control of sample use (45%). Professional background was significantly associated with both awareness (p = 0.0004) and willingness to donate (p = 0.0013). Logistic regression confirmed that respondents in medical or veterinary professions were more likely to support donation (odds ratio = 3.31, 95% confidence interval = 1.54–7.12, p = 0.002). No significant associations were found with age, gender, education, or religion.

**Conclusion:**

This first Baltic survey reveals that Latvian pet owners strongly support veterinary biobanking despite limited awareness. Altruism and the expected benefits of research drive participation, while ethical expectations regarding confidentiality, consent, and transparency remain crucial. The findings provide a foundation for developing national veterinary biobanking strategies, improving public communication, and integrating Latvia into European One Health and translational research infrastructures.

## INTRODUCTION

Veterinary biobanks are specialized repositories designed to collect, store, and manage biological samples and associated data from animals, particularly domesticated species. These resources support research, diagnostics, and education, thereby advancing veterinary medicine, animal health, and related disciplines [1]. Comparable to human biobanks established within hospitals and clinical networks, veterinary facilities play a central role in sample collection. However, while human biobanking infrastructures are well developed across Europe, veterinary biobanks remain scarce and underrecognized. According to the Biobanking and Biomolecular Resources Research Infrastructure – European Research Infrastructure Consortium (BBMRI-ERIC) directory, 403 organizations currently collect biological samples (https://directory.bbmri-eric.eu/ERIC/directory/#/catalogue), but only a limited number are dedicated to non-human specimens, with most focusing on infectious diseases and tumors in domestic animals. Examples include the Italian Biobank of Veterinary Resources at the Istituto Zooprofilattico Sperimentale della Lombardia e dell’Emilia Romagna, which conserves and distributes cell lines, microbial strains, and genomic resources from domestic animals [2]; the Instituut voor Tropische Geneeskunde (ITG) collection in Belgium, which maintains infectious disease and entomological samples from multiple regions [3]; the VetSuisse Biobank (VET_GEN_BERN) at the University of Bern, Switzerland, which archives blood, DNA, and tumor tissues [4]; and the VetBiobank at Vetmeduni Vienna, which has established a large-scale, high-quality tumor tissue collection, and reference archives from cats and dogs [5]. Outside the BBMRI-ERIC network, France hosts Centre de Ressources Biologiques pour les Animaux Domestiques, a national infrastructure coordinating the preservation of genetic and biological resources from domestic species to support breeding, veterinary research, and conservation [6]. Despite their limited number, these initiatives hold significant potential for advancing One Health, translational medicine, and the implementation of the replacement, reduction, and refinement (3Rs principle). In Latvia, however, no veterinary biobank has yet been established, and public perspectives on the subject remain unexplored.

Veterinary biobanks can reduce reliance on live animal experiments by providing access to samples representing a wide range of spontaneous diseases and disease stages [7–10]. With the human–animal bond continuing to strengthen globally, there is growing interest in improving companion animal health through scientific innovation [11]. Nevertheless, the sustainability of veterinary biobanking depends on active public engagement, as pet owners’ willingness to contribute samples is pivotal in shaping biobank practices. Lessons from human biobanking demonstrate that understanding donor motivations is crucial for enhancing recruitment and retention [12]. While many pet owners express openness to participation, concerns persist regarding consent, data protection, sample use, and perceived benefits [13, 14]. Altruistic motives, such as contributing to research that benefits future animals and humans, are strong drivers, alongside helping pets and families in similar circumstances, supporting veterinary professionals, and advancing scientific knowledge [13, 14]. Addressing these motivations and concerns through education and transparency is essential for building trust and ensuring the long-term success of veterinary biobanking.

Although human biobanking is well established across Europe, veterinary biobanking remains underdeveloped and underrecognized. Only a small number of repositories currently store non-human samples, most of which focus on infectious diseases or tumors in domestic animals. Examples include established infrastructures in Italy, Belgium, Switzerland, Austria, and France, but the Baltic region lacks comparable initiatives. Despite the recognized scientific value of veterinary biobanks in advancing One Health, translational medicine, and the 3Rs principle, there is a paucity of research examining public knowledge, acceptance, and ethical considerations in this domain. Existing studies, primarily from Western Europe and the United Kingdom, suggest that pet owners are motivated by altruism and scientific advancement, yet concerns regarding consent, data governance, and transparency remain prevalent. To date, no published studies have explored the awareness, motivations, and concerns of pet owners regarding veterinary biobanking in Latvia or the broader Baltic region. This gap limits the ability of policymakers, veterinary institutions, and researchers to design ethically grounded and socially acceptable frameworks for implementing veterinary biobanks in this geographic context.

The present study aimed to investigate the perspectives of pet owners in Latvia regarding veterinary biobanking. Specifically, it sought to assess (i) the level of awareness among Latvian pet owners, (ii) their willingness to contribute biological samples from both healthy and ill pets, (iii) the motivations driving participation, and (iv) the ethical concerns that may act as barriers. Furthermore, the study examined demographic and professional factors associated with awareness and willingness, to identify subgroups that could serve as early advocates for veterinary biobanking. By addressing these objectives, the study provides the first empirical evidence from Latvia and the wider Baltic region, offering a foundation for future development of veterinary biobank infrastructures, governance policies, and public engagement strategies that align with national and European research priorities.

## MATERIALS AND METHODS

### Ethical approval

The Animal Welfare and Defense Ethics Council of the Faculty of Veterinary Medicine, Latvia University of Life Sciences and Technologies (LBTU), reviewed the study design. As no interventions involving humans or animals were performed, no formal approval number was required. The study was conducted in accordance with the General Data Protection Regulation, Regulation (EU) 2016/679.

### Study period and location

This cross-sectional survey was conducted in Latvia between April 02 and May 09, 2025. The study period was chosen to align with the institutional veterinary biobanking project timeline and to maximize respondent accessibility during spring, when veterinary clinic attendance is typically high. Recruitment was carried out through social media platforms and in collaboration with veterinary clinics.

### Respondents

The target population consisted of Latvian residents who were current pet owners. A total of 166 responses were collected. During data cleaning, six inconsistencies were identified where respondents reported not owning pets but simultaneously indicated ownership of dogs or cats. Four of these cases were treated as mis-selections and retained, while two were excluded as genuine non-owners. The final dataset, therefore, included 164 confirmed pet owners (100%).

The survey link was distributed widely through multiple Facebook channels, including the official accounts of the Latvia University of Life Sciences and Technologies and its Faculty of Veterinary Medicine, private accounts, and animal-owner groups. Recruitment was further supported by three veterinary clinics: The state reference clinic at the Faculty of Veterinary Medicine in Jelgava, which serves a large patient population, and two high-traffic clinics in Riga. These clinics were chosen to capture a broad and diverse clientele. As recruitment was open and online, the total number of individuals reached could not be determined, and response rates were not calculated. The survey remained active for just over 1 month, closing with 166 complete responses after participation plateaued despite renewed social media promotion. No formal power calculation was performed, as this was an exploratory descriptive study. A target sample size of approximately 150–200 respondents was established based on feasibility and comparability with previous surveys on pet-owner perspectives toward veterinary biobanking [14].

### Questionnaire development

The questionnaire consisted of 49 items, divided into seven thematic sections (see Supplementary Table S1 for the full version and Supplementary Table S2 for a thematic summary). Its design was adapted from previously published surveys investigating pet-owner perspectives on biobanking and veterinary research participation [13, 14], with modifications to reflect the Latvian context.

A draft version was reviewed by academic colleagues, veterinary professionals, and members of the general public. Feedback from this informal pilot testing informed revisions aimed at improving clarity, structure, and relevance. The final questionnaire primarily used multiple-choice and true/false questions, with some items offering an “Other” option accompanied by a free-text field. The final item invited respondents to share additional comments or concerns regarding veterinary biobanking. The questionnaire was administered in Latvian, with an English translation prepared for publication.

### Data collection

Data were collected through a structured online questionnaire created with Google Forms (Supplementary Table S1). Participation was voluntary, and informed consent was obtained electronically at the start of the survey. The survey was fully anonymous, with no identifying information collected. Completed responses were exported to a password-protected Excel file accessible only to authorized members of the research team.

### Statistical analysis

Survey responses were exported from Google Forms to Microsoft Excel and analyzed using GraphPad Prism version 8.1 (GraphPad Software, La Jolla, CA, USA). The main outcome variables were as follows:

- Awareness of veterinary biobanking.
- Willingness to donate samples (from healthy or ill pets, DNA, and in euthanasia scenarios).
- Motivations (e.g., altruism, contribution to science, and expected treatment benefits).
- Concerns (e.g., confidentiality, withdrawal rights, and sample-use control).

Descriptive statistics (frequencies and percentages) were used to summarize demographics, awareness, motivations, and concerns. Associations between demographic characteristics (age, gender, education, religion, professional background, and type of pets owned) and both awareness and willingness to donate were tested using the Chi-square analyses. For subgroup analysis, religious affiliations were consolidated into four categories: Believer (Catholic, Lutheran, Orthodox, or other self-identified religious groups), non-believer (atheist and agnostic), unsure, and other.

A Bonferroni correction was applied to the Chi-square tests to account for multiple comparisons, with the adjusted significance threshold set at p < 0.0071. Effect sizes were reported as Cramer’s V. To further validate findings, a binary logistic regression was performed using prior awareness and professional background as predictors of willingness to donate in the healthy-pet scenario. Missing responses were excluded list-wise from each relevant analysis.

## RESULTS

### Respondent demographics

**Figure 1:**
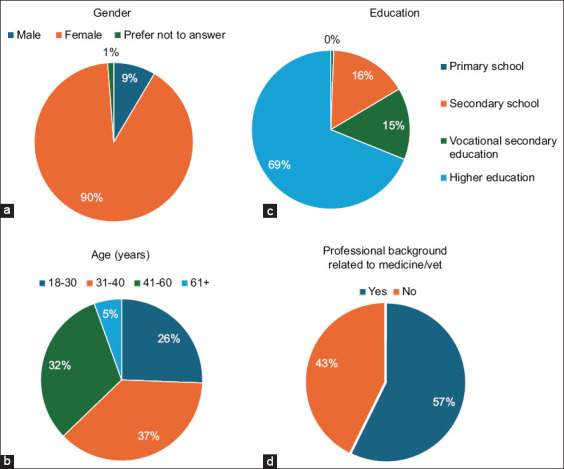
Key demographic characteristics of the 164 survey respondents Pie charts illustrate the distribution of respondents by (a) gender, (b) age group, (c) education level, and (d) profession related to medicine or veterinary medicine. The sample was predominantly female, with a high proportion of higher education and significant representation from medical/veterinary professionals.

A total of 164 pet owners were included in the final analysis. The majority of respondents were female (90.2%), while 8.5% were male (Figure 1a). Most respondents resided in Riga (30.1%) or other large cities (29.4%), with fewer from rural areas and small towns (21.5%) or other Latvian cities (19.0%). The largest age group was 31–40 years (37.2%), followed by 41–60 years and 18–30 years, whereas respondents over 60 years accounted for only 5.5% of the sample (Figure 1b). Educational attainment was high, with 68.9% reporting higher education, and more than half (57.3%) working in medicine or veterinary-related professions (Figures 1c and d). The most common religious affiliations were atheism (24.4%) and Lutheranism (18.3%) (Supplementary Figure 1). All respondents owned at least one pet. Dogs (69.5%) and cats (68.3%) were the most frequently reported species, though some respondents also kept farm animals, rodents, birds, or exotic pets. A detailed distribution is provided in Table S3.

### Awareness of veterinary biobanking

Awareness of veterinary biobanking was generally low. Of the 166 initial respondents, only 21.7% had heard of the concept before the survey, despite the sample’s high levels of education and professional involvement in human or veterinary medicine.

### Willingness to contribute to veterinary biobanking

Among the 164 confirmed pet owners, 67.1% expressed willingness to donate biological samples from a healthy pet; this figure rose to 75.6% when the pet suffered from a chronic or serious illness. Similarly, 75.6% indicated willingness to donate their pet’s DNA for research purposes. Only 7.2% reported prior enrolment of their pets in a clinical trial. Notably, participation in human clinical trials was strongly associated with pet enrolment in veterinary trials (p < 0.001). No significant differences were observed between dog and cat owners regarding willingness to donate (p = 0.640).

### Motivations for participation

The strongest motivator was the belief that research could benefit their own pet, family, or other animals (77.4%). A substantial proportion also highlighted the potential to improve treatment options (70.1%). More than half (56.1%) were willing to donate samples or organs in the event of humane euthanasia. Financial support for diagnostics or treatment increased willingness for 67.1% of respondents. Importantly, 89.8% expressed a desire to receive individual research results derived from their pets’ samples, indicating a strong interest in maintaining a connection with the research process.

### Concerns and ethical considerations

Respondents identified several concerns, primarily related to ethics and data governance. Almost all (90.9%) agreed that personal and pet-related data must remain confidential. In addition, 59.8% wished to retain the right to withdraw samples, and 44.6% wanted greater control over how samples would be used.

### Expectations and understanding of veterinary biobanking

A majority (74.4%) recognized that their pet’s biological samples might be shared with external researchers. Nevertheless, expectations regarding ownership and control persisted: 59.8% expected the right to withdraw samples, and 44.5% expected to decide how samples were used.

### Associations between demographics, awareness, and willingness

**Figure 2:**
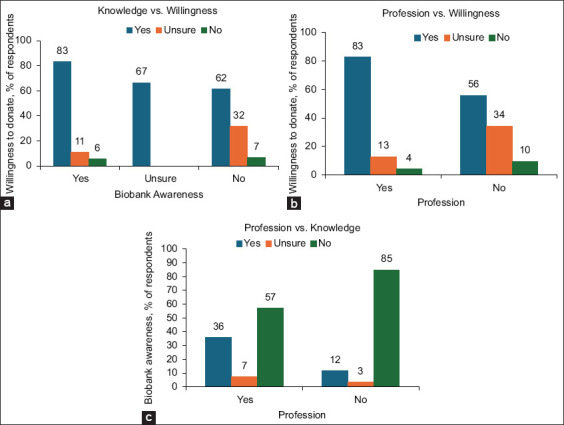
Relationships between biobank awareness, professional background, and willingness to donate pet samples. (a) Willingness to donate samples based on prior awareness of veterinary biobank. Compared with those who had not heard of them or were unsure, those who had heard of veterinary biobank were more likely to express a willingness to donate biological samples from their pets. (b) Willingness to donate samples by profession, where “profession” refers to whether respondents reported a background in human or veterinary medicine versus a non-medical field. Respondents with a background in medicine or veterinary medicine showed a greater desire to contribute samples, whereas non-professionals were more likely to be unsure or unwilling. (c) A significantly greater proportion of respondents with medical or veterinary backgrounds had heard of veterinary biobanks than those without such backgrounds. Only the associations in (b) and (c) remained statistically significant after the Bonferroni correction (adjusted threshold p < 0.0071).

Chi-square analysis revealed no significant associations between willingness to donate and gender (p = 0.907) or age (p = 0.688) (Table 1). Similarly, religious affiliation (p = 0.702) and education level (p = 0.635) were not associated with awareness or willingness (Table 1). Although prior knowledge of veterinary biobanking was linked to greater willingness (p = 0.017), this association was not significant after Bonferroni correction, and the effect size was small (Cramer’s V = 0.157) (Table 1, Figure 2).

**Table 1:** Associations between demographic variables, biobank awareness, and willingness to contribute to pet samples.

Comparison	Chi-square	df	p-value	Cramer’s V
Gender versus willingness	1.02	4	0.907	0.00
Age versus willingness	3.92	6	0.688	0.00
Knowledge versus willingness	12.01	4	0.017	0.157
Education versus knowledge	2.55	4	0.635	0.00
Religion versus willingness	3.81	6	0.702	0.00
Profession versus willingness	13.25	2	0.0013**	0.285
Profession versus knowledge	15.8	2	0.0004**	0.312

** Bonferroni-adjusted significance threshold = 0.0071. Only p-values below this threshold are considered statistically significant, df = Degree of freedom.

By contrast, a professional background was a strong predictor. Respondents with medical or veterinary training were both more aware of biobanking (p = 0.00037, Cramer’s V = 0.312) and more willing to donate (p = 0.0013, Cramer’s V = 0.285) (Table 1, Figure 2). Logistic regression further confirmed profession as an independent predictor of willingness (odds ratio [OR] = 3.31, 95% confidence interval [CI] = 1.54–7.12, p = 0.002), while prior awareness did not remain significant (OR = 2.06, 95% CI = 0.76–5.57, p = 0.153). The cross-tabulated results are presented in Table 2, illustrating the distribution of responses across key associations between knowledge, profession, and willingness.

**Table 2:** Contingencies for significant associations.

Knowledge of biobanks versus willingness to donate			

Knowledge	Willingness (%)		

	Yes	Unsure	No
Yes (n = 36)	30 (83.3)	4 (11.1)	2 (5.6)
Unsure (n = 6)	6 (66.7)	0	0
No (n = 119)	74 (61.7)	37 (31.1)	8 (6.7)

**Profession versus willingness to donate**			

**Profession**	**Willingness (%)**		

	**Yes**	**Unsure**	**No**

Yes (n = 70)	58 (82.9)	9 (12.9)	3 (4.3)
No (n = 93)	52 (55.9)	32 (34.4)	9 (9.7)

**Profession versus knowledge of biobanks**			

**Profession**	**Knowledge (%)**		

	**Yes**	**Unsure**	**No**

Yes (n = 70)	25 (35.7)	5 (7.1)	40 (57.1)
No (n = 93)	11 (11.8)	3 (3.2)	79 (84.9)

The cross-tabulated values show the distributions of the responses for knowledge versus willingness, profession versus willingness, and profession versus knowledge. Differences in totals are attributed to item-specific.

## DISCUSSION

### Main findings

This study is the first to examine the awareness, attitudes, and willingness of pet owners in Latvia to participate in veterinary biobanking. The results demonstrate a generally positive perception, with most respondents expressing a willingness to contribute biological samples, particularly in cases of chronic or serious illness in their pet. These findings align with those of previous studies [13, 14], confirming that altruism and a desire to support veterinary and translational research often drive pet owners.

To the best of our knowledge, no published studies have examined public perspectives on veterinary biobanking in the Baltic region or broader Central and Eastern Europe. This highlights the novelty of this research and underscores the need for more regional data to inform policy and public engagement strategies. Although only 22% of the respondents had prior awareness of veterinary biobanking, their willingness to participate was high once the concept was explained. This low awareness was observed despite the high educational background of many respondents, indicating that formal education alone does not ensure awareness of veterinary biobanking.

This highlights the need for enhanced public education and communication strategies to raise awareness and promote trust in the biobanking processes. Respondents frequently cited potential benefits to their pets, families, and other animals as key motivators. In addition, the expectations surrounding data confidentiality, the right to withdraw, and the transparency of sample use highlight areas where veterinary biobanks should focus their ethical and procedural efforts.

### Implications and relationship with previous studies

The respondents also expressed a desire to receive relevant research outcomes, demonstrating their interest in remaining connected to the scientific value of their contribution. A notable proportion of respondents expressed interest in receiving individual research results derived from their pet’s biobank samples (89.8%). The unexpectedly high demand for return of results observed in this study introduces a novel ethical consideration for veterinary biobanking and raises important questions about whether veterinary biobanks should adopt human-style feedback policies for owners.

This reflects the growing expectations of research participants for transparency and feedback, a trend well-documented in human biobanking [15, 16]. In the veterinary context, this interest may be linked to a desire to actively contribute to science and remain informed about how their pet’s contribution is used. Importantly, this expectation coexisted with strong altruistic motivations, with many respondents emphasizing the value of contributing to research that could broadly benefit other pets, families, or society. This combination of altruism and desire for personal feedback reveals a dual motivation that represents a novel insight in the field of veterinary biobanking.

Veterinary biobanks are becoming integral to translational research efforts. For example, similar to human breast cancer cells, canine mammary tumor organoids are sensitive to drugs, suggesting that they could be valuable tools for preclinical drug testing [17]. Moreover, biobank organoid cell lines can be subcultured indefinitely, reducing the need for continuous tissue harvesting [17, 18]. These models offer a powerful preclinical tool for both veterinary and human drug development, and their existence depends on access to well-characterized, ethically sourced tissue.

Owners who contribute samples may feel a stronger connection to such research and thus value receiving updates or relevant findings, especially if they can impact treatment decisions. However, the return of results in veterinary settings raises logistical and ethical challenges. Delivering validated, clinically relevant findings requires additional resources for laboratory confirmation, professional interpretation, and communication with owners, which may substantially increase costs. There is also potential for misinterpretation or anxiety if findings are ambiguous or lack clinical relevance. Unlike human participants, animals cannot provide informed consent, adding a further ethical layer to such feedback.

At present, there are no standardized protocols for returning results to veterinary biobanks. To address these concerns, biobanking frameworks should carefully define which types of results, if any, are appropriate to return and establish processes that involve veterinary professionals in communicating findings where needed. Clear policies are crucial for striking a balance between owner engagement, scientific responsibility, financial feasibility, and animal welfare considerations.

### Alignment with the aims of veterinary biobanking

These motivations are well-aligned with the broader aims of veterinary biobanking, which include improving companion animal health, enabling ethically sourced research materials, and supporting the 3Rs principle of live animal use. By leveraging clinical samples collected during routine care or treatment, veterinary biobanks reduce the need for dedicated experimental animals while still enabling high-quality research.

Recent advances underscore the scientific potential of these resources. For example, biobanks provide access to samples collected from various disease stages, particularly from animals involved in longitudinal studies, which can offer critical insights into disease progression. Longitudinal biobanking initiatives, such as the Dog Aging Project, the Senior Family Dog Project, the Golden Retriever Lifetime Study, and the Mars Petcare Biobank, enable the study of the onset and progression of conditions, such as aging, cancer, and cardiovascular diseases in companion animals [7, 19, 20]. Furthermore, projects such as the Large-scale Unified Project for Canine Genetics (LUPA) have shown how biobanks can pool resources across institutions and countries to support complex genetic studies [21].

### Broader scientific and policy relevance

Biobanks also promote the One Health approach by supplying valuable samples from spontaneous disease models found in companion animals, facilitating cross-species research and improving the understanding of zoonotic disease transmission and antimicrobial resistance. Since nearly 75% of emerging human infections are zoonotic [22], companion animals, which share close environments with humans, have the potential to act as sources and sentinels of a broad spectrum of zoonotic infections [23].

The SAVSNET and VetCompass in Australia provide crucial insights into disease dynamics, transmission risks, and antimicrobial usage patterns, highlighting the relevance of veterinary biobanks in supporting One Health surveillance and public health strategies [24–29]. Positioning veterinary biobanking within the One Health framework highlights its role in comparative medicine and enhances zoonotic disease surveillance, thereby supporting the integration of veterinary and human health infrastructures in Europe. These advantages make animal-tissue biobanks powerful tools for advancing scientific knowledge in an ethical, cost-effective, and collaborative manner.

### Subgroup insights

The subgroup analysis revealed an important context for understanding the factors that influence owner engagement with veterinary biobanking. While attitudes toward participation were broadly consistent across gender, age, and education level, key factors emerged as awareness and professional background. Although prior knowledge of biobanking was initially associated with greater willingness to participate (p = 0.017), this relationship did not remain statistically significant after a Bonferroni correction was applied for multiple comparisons (adjusted threshold p < 0.0071). Nonetheless, the observed trend supports the hypothesis that increasing public understanding can positively influence engagement.

Comparable findings in the United Kingdom have reported willingness to participate between 65% and 89% despite relatively low awareness [13, 14, 30]. Our Latvian findings (healthy 67.1%, ill 75.6%, and awareness 22%) are consistent with these results, indicating that while awareness remains limited, willingness to engage is robust once the concept is explained.

A professional background also played a significant role. Respondents working in medical or veterinary fields demonstrated greater awareness and willingness to participate, suggesting that familiarity with clinical research environments promotes a higher acceptance of biobank initiatives. These professionals can serve as important advocates in promoting participation among the broader public. Moreover, individuals who had participated in human clinical trials were more likely to report pet participation in veterinary research, indicating a link between personal experience and openness to biobank involvement.

Overall, the results underscore the importance of targeted communication strategies in enhancing awareness and clarifying the purpose and benefits of veterinary biobanking, particularly in fostering trust and increasing participation among pet owners who are not directly affiliated with scientific or medical professions.

### Gender dynamics and sociocultural factors

The respondent population in this study was predominantly female, a trend observed in human health research and several survey-based studies involving pet owners [13, 14, 30]. This likely reflects broader societal patterns, where women often assume a primary role in caregiving and health decision-making for family members and companion animals [31]. Women are more likely to participate in surveys and engage in health-related discussions in human health research, although they remain underrepresented in some areas of clinical research participation [32, 33].

In our study, statistical analysis revealed no significant association between gender and willingness to donate biological samples, suggesting that gender did not influence attitudes toward veterinary biobanking in this context. As the sample was strongly female-dominated, the statistical power to detect gender-related differences was limited. Therefore, potential gender effects cannot be excluded and should be examined in future studies with more balanced samples. The absence of data on the sex of the animals represents another limitation. Future studies should consider whether owners’ attitudes differ based on their sex, particularly for reproductive tissues or postmortem donations, which may carry different ethical or emotional considerations for some owners.

The final section of the survey invited open-ended responses for any remaining thoughts or concerns. One respondent reflected broader societal concerns by commenting that veterinary biobanking may be perceived as premature in a country where issues such as pet registration and animal welfare infrastructure remain unresolved. This statement reflects the view that systemic issues, such as unregulated pet ownership and inadequate animal registration, should be addressed before initiating advanced research initiatives, including biobanking. This highlights the significance of public perception and social trust, underscoring the importance of framing biobanking as complementary rather than competing with basic animal welfare infrastructure.

These perspectives highlight barriers that extend beyond owner willingness, including gaps in veterinary infrastructure, governance, and regulatory oversight. Addressing such challenges through investment, ethical frameworks, and clear communication is critical for ensuring the long-term sustainability and public credibility of veterinary biobanking in Latvia.

### Limitations

This study has several limitations. The sample may not fully reflect the wider Latvian population, as recruitment was primarily conducted online and through veterinary clinics in Jelgava and Riga, which may underrepresent rural areas. This recruitment approach may have introduced sampling bias, potentially overrepresenting respondents with higher education, better internet access, or a professional connection to animal health services.

As recruitment was open, the total number of individuals reached was unknown, and no precise response rate could be calculated. The sample was strongly female-dominated, which limited the ability to identify potential gender-related differences. Socioeconomic variables (e.g., income, occupation categories beyond medical/veterinary) and detailed pet information (e.g., age, breed, health status, vaccination, or insurance) were not collected to reduce the respondent burden, as the questionnaire already contained 49 items.

The self-selected nature of participation could also contribute to response bias, favoring individuals with a greater interest in research or veterinary topics. In addition, the questionnaire was conducted in Latvian, which could have excluded minority-language speakers, further limiting generalizability. Finally, the analyses were restricted to dog and cat owners; exotic pet owners were too few for stratified analysis.

These limitations are common in exploratory survey studies but should be addressed in future research through broader recruitment, inclusion of minority groups, and collection of socioeconomic and pet-level data.

### Future directions

Although this study is exploratory and reflects early adopter attitudes, it offers important insights for shaping veterinary biobanking efforts in Latvia. These findings may serve as a foundation for the development of future veterinary biobank infrastructure and public engagement strategies.

As awareness remains low, targeted educational initiatives, particularly through trusted veterinary professionals, could help introduce biobanking as a valuable part of clinical and research practice. Although Latvia does not currently have a national veterinary biobank system, the initiative underway at the Faculty of Veterinary Medicine of the Latvian University of Life Sciences and Technologies represents a first step toward establishing a national veterinary biobank system.

This study also demonstrates a shift from researcher-driven goals toward pet-owner–centric governance, suggesting a model for community-engaged veterinary biobanking that could be applied in other countries. Unlike in human biobanking, where individuals can consent for themselves, veterinary biobanking faces the additional ethical complexity that pets cannot provide consent; thus, owner expectations regarding transparency and the return of results become especially critical.

These early insights can inform the design of governance frameworks, community engagement models, and ethical practices that prioritize transparency, trust, and integration with companion animal healthcare priorities. In the future, integration with BBMRI-ERIC and cross-Baltic collaborations will be crucial to ensure sustainability, harmonization with European standards, and contribution to One Health surveillance.

## CONCLUSION

This study presents the first systematic evaluation of pet owners’ perspectives on veterinary biobanking in Latvia and the broader Baltic region. Although only 22% of respondents had prior awareness of veterinary biobanks, the majority expressed a strong willingness to donate biological samples, particularly when their pets were affected by chronic or serious illness. Altruistic motivations, such as helping other animals and contributing to scientific research, were consistently identified as key drivers, while concerns centered on confidentiality, withdrawal rights, and transparency regarding the use of samples. Notably, almost 90% of respondents expressed an interest in receiving individual research results derived from their pet’s samples, revealing a dual motivation of altruism coupled with a desire for personal engagement in research.

The findings have several practical implications. Veterinary professionals and policymakers should prioritize transparent communication strategies that clarify the purpose, governance, and benefits of veterinary biobanking. Addressing owner concerns about data protection and consent is critical to building trust and ensuring sustainable participation. The significant association between professional background and willingness to donate suggests that veterinary and medical professionals may play a crucial role in advocating for the wider public to engage. Furthermore, integrating veterinary biobanking into the One Health framework can strengthen surveillance of zoonotic diseases, advance translational research, and reduce reliance on live animal experimentation through the 3Rs principle.

A key strength of this study is its originality: It represents the first exploration of veterinary biobanking attitudes in Latvia, providing baseline evidence for future national and regional initiatives. The diverse recruitment channels and relatively large sample size further enhance its relevance. However, limitations such as the female-dominated sample, online recruitment bias, and exclusion of minority-language speakers should be acknowledged. Future studies should aim for broader representation and inclusion of socioeconomic and pet-level data.

Latvian pet owners demonstrate strong support for veterinary biobanking, despite having a low baseline awareness. Their perspectives underscore the importance of education, ethical governance, and transparent feedback systems in shaping the practices of biobanks. These early insights provide a foundation for developing a national veterinary biobank in Latvia, aligning with European infrastructures such as BBMRI-ERIC, and advancing the role of veterinary biobanking in One Health, translational research, and companion animal health care.

## DATA AVAILABILITY

The anonymized dataset generated and analyzed in the present study is available from the corresponding author on reasonable request.

## AUTHORS’ CONTRIBUTIONS

GS, KK, and LK: Conceptualized the idea and edited and drafted the manuscript. GS: Collected, analyzed, and interpreted the data and wrote the manuscript. All authors have read and approved the final version of the manuscript.
